# Gaming motivation and well-being among Norwegian adult gamers: the role of gender and disability

**DOI:** 10.3389/fmedt.2024.1330926

**Published:** 2024-04-11

**Authors:** Beate W. Hygen, Christian Wendelborg, Bård Erlend Solstad, Frode Stenseng, Mari Bore Øverland, Vera Skalicka

**Affiliations:** ^1^ Department of Diversity and Inclusion, NTNU Social Research, Trondheim, Norway; ^2^ Department of Sport Science and Physical Education, University of Agder, Kristiansand, Norway; ^3^ Department of Education and Lifelong Learning, Norwegian University of Science and Technology, Trondheim, Norway; ^4^ Department of Psychology, Norwegian University of Science and Technology, Trondheim, Norway

**Keywords:** gaming, gender, motivation, network, disability, well-being

## Abstract

**Introduction:**

Digital gaming is a popular and often social activity, also among adults. However, we need more knowledge of the social dynamics of gaming and its potential benefits for one's well-being. The current study aimed to examine gaming motivation, time spent gaming, and gaming performed together with friends, family, or romantic partner and how these aspects relate to expanded social network and well-being among men and women with and without disability.

**Methods:**

Regular players of the popular game Fortnite Battle Royale (FBR; *N* = 278, 48.5% women, Mage = 32.38) completed an online questionnaire assessing their motivations for playing FBR (social motivation, achievement motivation, novelty motivation), time spent gaming, whom they usually play with, their psychological well-being, and FBR's impact on their life and social network. Differentiated statistical analyses on gender and disability were performed.

**Results and discussion:**

The results showed that time spent gaming and social motivation to play were associated with larger social networks for all participants (strongest for women). More time spent gaming FBR was also associated with a positive impact on life for those with a disability. Social motivation to play was positively associated with a positive impact on life for men and those without a disability and increased well-being for women. Novelty motivation, which concerns experiencing new features in the game, was associated with a positive impact on life for women and with a decrease in well-being for those with a disability. This study demonstrated that gaming can be an essential social arena associated with positive outcomes for men, women and disabled people, who—when socially motivated—may expand their social networks through gaming.

## 1 Introduction

Gaming is an activity that appeals to people of nearly all ages (the average age of a gamer is 33 years (1);, genders [48% is female (1)], and over three billion people around the world are active players (2). Despite this popularity, gaming researchers, especially in psychology, have focused on the negative outcomes of gaming (3–9). However, in recent years, more attention has been devoted to positive gaming outcomes such as cognitive benefits (10, 11). Still, there is a need for more knowledge concerning the potential benefits of gaming, especially with respect to social benefits and well-being, although some research exists (12, 13). A systematic literature review of 18 studies showed that engaging in Massively Multiplayer Online games was positively correlated with social well-being (14), whereas another review highlighted gaming motivation as an important moderator of the impact of gaming on well-being (15). Despite these efforts, we lack knowledge regarding for whom gaming can be particularly beneficial from a well-being perspective. A recent critical review (16) stated that “unless the social context (who), type (what), motivation (why), time and day (when), and amount (how much) of video gaming activities are adequately considered, examinations of well-being outcomes in relation to video gaming will remain incomplete” (p. 1). In the current study, we aim to address these shortcomings by including most of these aspects (except the question of *when*). Additionally, we will examine different associations between gaming-related factors (e.g., gaming motivation, time spent gaming), and whether potential outcomes differ for men, women, and those with or without a disability to answer the question *for whom*.

Socializing is an important motivation for gaming (17) and many online games are designed so that many players must work together to achieve goals in the game. In many cases, that requires the players to communicate substantially with each other (verbally or by chat functions). This also applies to battle royale games such as Fortnite Battle Royale (FBR) due to their reliance on team-based play (when not played in solo mode). Games may likely facilitate new friendships and extended networks (1), but we know less about how and to whom this may be the case.

A few studies have examined the relation between gaming and well-being, but there is a great variety in samples and measures (12, 13, 18, 19), and thus the results are mixed, as some studies show a positive relationship (18), whereas others show a negative relationship (12). Nevertheless, some evidence shows that gaming can repair negative mood (20). It has been argued that the influence of gaming on well-being is moderated by factors such as social interaction while gaming and the motivation individuals report for gaming (15), and these arguments have been supported empirically. For instance, a recent study (21) identified two subgroups of heavy gamers: the social (those who exhibited more online social interaction) and the non-social, and found differences in well-being between these two groups. More specifically, being in the heavy non-social group was associated with lower well-being. Gender differences were also found; non-social male gamers reported more social anxiety, while social female gamers reported less social anxiety and loneliness. In another study (consisting of Pokémon players), playing for fun and friendship maintenance were positively associated with increased well-being, while escapism and nostalgia motivations were negatively associated with well-being (22).

There is no doubt that socialization and relationships are vital for good mental health (23), and it may be that the social side of gaming is more vital and beneficial for some individuals compared to others. According to the *need-to-belong hypothesis* (24), humans must establish and maintain relationships with others. Individuals who, for various reasons (i.e., having a physical or psychological disability), have limited access to others may seek out alternative arenas to fulfil their need to belong. Gaming may be such an arena as communication is easily accessible; one can connect with others from home and reach out to many people with common interests with little effort. Moreover, gaming is an activity where participation is on “equal terms” and provides individuals with an identity defined by the gaming activity rather than being disabled, and may offer a new arena for self-presentation (25); in gaming, you “are just like any other gamer”.

Motivation for gaming has been understood through self-determination theory (SDT) (26), postulating three basic psychological needs that must be fulfilled to be intrinsically motivated: autonomy, competence, and relatedness. Previous studies have shown an association between satisfaction of the three basic psychological needs and more time spent on gaming (27) and how much players enjoy the game (28, 29). Thus, many may game to experience a sense of competence and autonomy, also overlapping with the need-to-belong assertion to feel socially related to others (24). It has been argued that people with various types of disability who suffer from limited mobility or experience fewer social interactions due to their impairment may be more inclined to game to satisfy their need for relatedness (30). There is scarce research on gaming and people with various disabilities, although some exist (25, 31, 32). We hypothesize that individuals with disabilities (physical and psychological) who are socially motivated to game will report higher levels of well-being compared to those socially motivated without a disability because the gaming arena may be one of the places to socialize for those individuals with a disability.

Furthermore, previous research demonstrates gender differences in gaming-related issues. For instance, fewer women play digital games compared to men, and there are gender differences in gaming motives and preferred gaming genres (33). Moreover, the effects of gaming have proven different depending on gender (34, 35). Women typically have more extensive social networks and friends than men (36). Thus, women may turn to gaming for social reasons (e.g., games to interact with others) more so than men. Such reasoning has been supported empirically (37). Perhaps this means that women are more inclined to increase their social networks through gaming compared to men when they are socially motivated to game. Alternatively, one may argue that socially motivated men may increase their network since gaming may be a more integrated part of boys' upbringing than girls' (35), and gaming may be a well-known arena for them to establish new friendships. We have no clear hypothesis concerning gender other than that it is plausible that women and men who are socially motivated for gaming may report expanded social network. Finally, we hypothesize that social motivation, not achievement and novelty motivation, is the strongest motivation predictor associated with positive social and emotional outcomes.

To address the research gaps raised by the mentioned review article (16), we examine whether time spent gaming FBR, different motivations for gaming, who one plays with, and to whom this may apply is associated with expanded network, impact on life, and well-being, and whether the associations differ according to gender or disability status.

## 2 Method

### 2.1 Study design

The present work is derived from the Norwegian Adult Gamer Study (NAGS), which is a survey study of adult gamers. Information from teen and adult gamers of FBR was obtained from 22. April to 29. April 2022. Participants received a detailed description of the study and were informed they could withdraw their consent anytime. The study design and recruitment has been described in a previous report (38).

### 2.2 Participants, recruitment and procedure

Participants were recruited through social media sites (i.e., Facebook, Instagram, Twitter, and Discord) targeting gamers of FBR. We recruited 278 participants over the age of 16 (49.1% women, *M*_age _*= *32.28 years, *SD *= 9.22). Age ranged from 16 to 63, with 11% under the age of 20, 26% age 20–29 years, 41% 30–39 years, and 21% age 40 or older. Three participants were identified as non-binary or as “other” regarding gender. These three were omitted from gender-specific analyses. Participants received no compensation for their participation in the survey, but they were in the draft to win three gift certificates of 500 Norwegian Kroner (approximately 50 American dollars) each. The Norwegian Agency for Shared Services in Education and Research recommended the project.

In the current study, we focus on players of FBR (the mode battle royale), which is one of the most popular battle royale games globally, with a player count of approximately 83 million (39). Players can communicate verbally (using a microphone), through in-game signs and chat options. FBR allows solo plays, duos, trios, and squads. Thus, players can decide how many they want to play with in each game round, and they can decide to play with friends or be assigned to random teams. FBR has a relatively simple plot; 100 players are dropped on an island, and the purpose is to be the sole survivor. Additionally, players can take on various quests during gameplay, which may facilitate cooperation with others. Moreover, FBR is well known for its humor (40). In this manner, FBR differentiates itself from many other action games, such as Counter Strike (CS-GO), which do not have the same humoristic elements. Humor promotes social bonding (41). Thus, these features make FBR a highly social game that may appeal to a wide variety of players (across different ages, genders, and social backgrounds). Based on the various ways one can play FBR, and the social elements that are somewhat different from other shooter games (as mentioned), we developed a new scale measuring the specific elements in FBR that may motivate the players (see below for further description).

### 2.3 Measures

#### 2.3.1 Expanded social network (ESN)

Expanded Social Network (ESN) was measured by the item “To what degree do you agree with the following statement: I have expanded my social network (formed new acquaintances, gained new friends who I meet regularly online or offline) through playing Fortnite Battle Royale”. The item was rated on a 4-point Likert scale: (1) *disagree*, (2) *somewhat disagree*, (3) *somewhat agree*, and (4) *agree*. For the analyses, we recoded the scale, making it dichotomous (1 and 2 were coded as *disagree* (*1*), and 3 and 4 as *agree* (*2*)). This question was a study-specific question developed by the current study's authors.

#### 2.3.2 FBR's impact on life

FBR's impact on life was measured by the item “What kind of influence has playing Fortnite Battle Royale had on your life?”. Participants answered on a 7-point Likert scale: (0) *very negatively* and (6) *very positively*. We acknowledge that this is a general question. However, we included it because we wanted to examine the subjective experience of whether participants experienced playing FBR as inherently “good” or “bad” influence on their lives. This question was a study-specific question developed by the current study's authors.

#### 2.3.3 Well-being

Well-being was measured by the Scale of Positive and Negative Experience (SPANE) (42). Participants were instructed to think about the last month and answer how often they had experienced positive and negative feelings on a five-point scale ranging from 1 *(rarely or never*) to 5 (*very often or always*). Negative feelings items were, for example, “*I have felt negative*” and “*I have felt sad*”. Positive feelings items were, for example, “*I have felt positive*” and “*I have felt happy*”.

To create a total score for well-being, we calculated the means for positive and negative feelings. Then, we subtracted the negative feelings' mean score from the positive feelings' mean score [SPANE-B (42)]. Thus, a higher score indicates positive well-being. The theoretical range is between −24 to 24. A reliability analysis provided satisfactory results (Cronbach's alpha of 0.92). The scale has been validated in several European studies (43–45).

#### 2.3.4 Total hours game time FBR

Participants reported how many hours they played FBR in a typical week. We reminded participants to include hours spent the entire week (weekdays and weekends) and provided an example to prohibit possible misunderstandings. For example, If you play an average of 3 h per day on weekdays (Monday to Friday) and 5 h on average per weekend days (Saturday and Sunday), you should report 25 h.

#### 2.3.5 Gaming motivation

Gaming motivation Was measured by The Fortnite Motivation Scale (FMS), which was inspired and developed by the authors, building on a study that found three overarching motivation components (achievement, social, and immersion) for playing MMORPGs (17). Inspired by some of these components, we developed a scale of 22 items adapted to fit FBR. These items were constructed to measure different motives to play FBR. The scale yielded three different types of gaming motivation: social, novelty and achievement (for further details concerning this instrument, see Results section).

#### 2.3.6 Gaming with family and friends

Participants were asked if they played FBR with their friends, family, or partners (individuals they socialize with offline and online). The item was rated on a 5-point Likert scale: (1) *never,* (2) *rarely,* (3) *sometimes,* (4) *usually*, and (5) *always*.

### 2.4 Disability

Participants were given the following definition of disability used in the Norwegian Labour Force survey: “Disability means long-standing health problems that can lead to limitations in daily life. It could be, for example, impaired vision, hearing, or mobility, reading and writing difficulties, heart or lung problems, mental disorders, and so on.” Based on this definition, the participants were asked: “In your opinion, do you have a disability?” Sixty participants (21.7%) answered yes to this question.

### 2.5 Age, work status, cohabitation status, game time, other games

Participant's ages were divided into four groups: Under 20 years, 20 to 29 years, 30 to 39 years and 40 or older.

Participant work status was coded into four groups: *full-time, part-time, no work affiliation* (e.g., unemployed, on disability benefits), and *in education* (student). Participants' living situation was coded into five groups: *living alone, living with someone* (e.g., roommates, parents), *living with children but no spouse* (single parents), *living with spouse but no children*, and *living with spouse and children*.

Participants reported total hours per week they played games other than FBR in the same manner as they reported time spent on FBR (see above).

## 3 Data analysis

The STATA software package version 17.0 Special Edition (46) was used for statistical analysis.

### 3.1 Factor analyses

We used a principal component analysis (PCA) as a preliminary analysis to establish the factors (components) in The Fortnite Motivation Scale (FMS). Further, we used confirmatory factor analysis (CFA) within the Structural Equation Modeling framework to assess the validity of the scales. The CFA analyses were used to verify the factor structure of observed variables and to test the hypothesis that relationships exist between observed variables and an underlying unobserved (latent) construct, and indicate the degree to which the measurement model in SEM is a good fit to the observed data (47). Regarding model fit, numerous goodness-of-fit indicators exist to assess a model: the Tucker-Lewis Index (TLI), Comparative Fit Index (CFI), Standardized Root mean square residual (SRMR), and root mean square error of approximation (RMSEA). The current study used the following criteria to determine model fit: TLI and CFI ≥ .95; SRMR and RMSEA ≤ .08 (48). After determining the fit of the scales, we then measured Raycov composite reliability (CR) to estimate the reliability within the CFA framework and internal consistency (reliability) using Cronbach's alpha. The CR is a credible alternative to Cronbach's alpha, which does not directly measure whether the indicators change on a single factor (49) and is commonly seen as more accurate than the alpha (50). It is recommended that the CR and Cronbach's alpha value have a minimum level of .70 (50). Higher values equal better internal consistency of the variables included in the scale.

### 3.2 Regression analyses

We applied twelve multiple regression analyses (one for each dependent variable separately for men, women, disabled, and not disabled) where all variables (including control variables: age, work status, cohabitation status, game time other games) were entered simultaneously in the models to analyze the association between the dependent (expanded network, impact on life, and well-being) and independent variables (e.g., total hours game time, gaming motivation, gaming with family and friends). When conducting disability-specific analyses, we controlled for gender. Descriptive bivariate analysis was conducted using Pearson's *r*, Kendal's tau-*b*, *t*-test, ANOVA, Cohen's *d*, Chi-square, and Cramer's V to gauge the difference between groups or relation between variables.

While the convention is to carry out logistic regression for binary variables, as is the case for the dependent variable Expanded Social Network, there are several limitations when using logistic regression with our dataset. First, we cannot interpret odds ratios as effect measures, as they also reflect the degree of unobserved heterogeneity in the model (51). Second, we cannot compare odds ratios for similar models across groups or with different independent variables (51). Both conditions are present in the models in our study. Standardized beta coefficients in OLS regressions are more useful than odds ratios when comparing estimates between groups and models. However, in cases where one wants to compare effect sizes across groups, the dependent variables must be continuous (52). In short, we are in a situation where we want to compare estimates between groups and models, but we, strictly speaking, do not have the data to do so. However, previous studies have concluded that there is limited practical difference between logistic and OLS regression. Therefore, it has been recommended to carry out standard OLS regression analysis (53), and we chose to use standardized effects in OLS regression to compare estimates between groups and models.

Both OLS and logistic regression were initially carried out for this study, confirming limited differences, and the same patterns were identified through either approach. However, it is essential to note that the specific estimates must be interpreted with caution.

## 4 Results

### 4.1 Fortnite motivation scale

A principal component analysis (PCA) yielded three factors: social motivation, novelty motivation, and achievement motivation. All items were phrased with reference to the sentence “What motivates you in Fortnite Battle Royale….”. The six items measuring social motivation focused on interacting with other players, being social, and getting to know others, e.g., “… getting to know new people”, “…cooperate with others (be on a team working together),” and “… talking and interacting with others (communicating with teammates)”. Novelty motivation consists of five items and focuses on motivation concerning experiencing new features in the game, such as: “…that there will be new seasons/updates”, “…to discover new places on the map/game”, “…to get new avatars, skins, emotes, backpacks and other accessories”. Four items measured achievement motivation which focused on the element of competition and improving in the game: “…to get better at the game (have progression)”, “…to get or increase the number on the victory crown” (a crown that symbolizes how many wins you have in a row), “…to take out other players (kills)”, “… to win.” The participants answered each item on a 4-point Likert scale ranging from 1 (*disagree)* to 4 (*agree)*.

We employed a confirmatory factor analysis through Structural Equation Modeling (SEM) to assess the validity of the three scales all of which yielded acceptable and satisfactory results: Social motivation: (TLI) = 0.99, (CFI) = 0.97, SRMR = 0.031, RMSEA = 0.063. All factor loadings were significant, and the reliability analysis provided satisfactory results (Raykov's Composite reliability of 0.77 and Cronbach's alpha of 0.79). Novelty motivation: TLI = 0.98, CFI = 0.99, SRMR = 0.023, RMSEA = 0.052. Again, all factor loadings were significant, and the reliability analysis provided satisfactory results (Raykov's Composite reliability of 0.86 and Cronbach's alpha of 0.83). Achievement motivation: TLI = 0.97, CFI = 0.99, SRMR = 0.028, RMSEA = 0.068. Like the previous scales, all factor loadings were significant, and the reliability analysis provided satisfactory results (Raykov's Composite reliability of 0.73 and Cronbach's alpha of 0.73).

### 4.2 Descriptives

Descriptives of the study variables are displayed in Table 1, which shows that approximately 22 per cent of the sample report having a disability, nearly 70 percent live with a spouse (with or without children), and nearly half of the sample works full-time.

**Table 1:** Descriptives of study variables.

	*N*	%			
Gender					
Male	140	50.9			
Female	135	49.1			
Disability status					
No disability	216	78.3			
Disability	60	21.7			
Work status (fulltime ref.)					
Fulltime	134	48.7			
Parttime	26	9.5			
No work affiliation	63	22.9			
In education	52	18.9			
Living situation					
Living with alone	34	12.2			
Living with someone	45	16.2			
Living with children. no spouse	32	11.5			
Living with spouse. no children	48	17.3			
Living with spouse and children	119	42.8			
	*N*	Mean	SD	min	max
Expanded Network	276	0.63	0.48	0	1
Impact on life	277	4.00	1.43	0	6
Well-being	271	8.41	8.32	−19	24
Total hours game time Fortnite (TGF)	278	13.47	9.99	1	50
Social motivation	277	3.39	0.59	1	4
Achievement motivation	275	3.27	0.62	1	4
Novelty motivation	272	3.05	0.77	1	4
Gaming with family and friends	272	66.34	33.95	0	100
Age (40 or older ref.)	278	32.28	9.22	16	63
Total hours game time other games (TGG)	278	15.82	13.65	0	90

Descriptives of the study's predictors by gender and disability status are displayed in Table 2. Notably, the disabled group showed the most time spent gaming FBR (on average 17.74 h per week), followed by women (15.61 h per week). Total game time per week (attained by adding the variables “Total hours FBR” and “Total hours game time other games”) was on average 35.44 for those with a disability, and approximately 29 h for women and men. Thus, in this sample, those with a disability (*M *= 35.44, *SD *= 18.63) played significantly more than those without a disability (*M *= 27.68, *SD *= 18.67), *t* (−2.85 = (274, *p *= .005). Moreover, people with disabilities displayed, on average, higher mean scores on social motivation; men scored highest on achievement motivation, and women scored highest on novelty motivation. Women were the ones who reported to game, on average, most with family and friends.

**Table 2:** Descriptives of the study's gaming variables by gender and disability status.

Predictors	Men			Women			No disability			Disability		
	M	SD	*N*	M	SD	*N*	M	SD	*N*	M	SD	*N*
Total hours FBR^a^	11.26	8.85	140	15.61	10.57	135	12.37	9.78	216	17.74	9.72	60
Total hours game time other games	17.84	14.20	140	13.50	12.75	135	15.32	12.99	216	17.70	18.83	60
Social Motivation	3.26	.65	139	3.52	.49	135	3.33	.61	215	3.59	.46	60
Achievement Motivation	3.30	.59	138	3.24	.65	134	3.26	.61	213	3.28	.67	60
Novelty Motivation	2.78	.84	136	3.32	.58	134	3.00	.78	212	3.21	.74	58
Gaming with family and friends	3.62	1.13	139	3.85	.93	135	3.74	1.00	215	3.75	1.19	60

^a^ FBR = Fortnite battle royale.

Descriptives of and group differences on the three outcome variables, Expanded network, Impact on life, and Well-being, are displayed in Table 3. There were no significant differences between participants regarding Expanded network. Some significant differences emerged for the dependent variable FBR impact on life; Women scored significantly higher than men, and age groups 30–39 years and 40 years and older scored significantly higher than those under 20 years. Those living with children (with and without a spouse) scored significantly higher than those living with someone (e.g., roommates). In this sample, those without a disability demonstrated significantly higher well-being than those with a disability, and those with no work affiliation showed significantly lower well-being than those who worked full-time. Finally, those living alone scored significantly lower on well-being than those living with a spouse and children.

**Table 3:** Distribution of background and outcome variables. Reporting *n* and percent for background variables; mean, standard deviation, Cohen's *d* and *f*-value on continuous outcome variables and percent, chi square and Cramer's V for dichotomous outcome variable.

	Expanded network			FBR impact on life			Well-being		
	% Yes	Chi	V	Mean	SD	Cohens *d*	Mean	SD	Cohens *d*
Gender									
Male	58.7	1.85	0.08	3.83	1.41	0.26*	9.36	7.27	0.20
Female	66.6			4.20	1.40		7.70	8.93	
Disability									
No	61.4	0.44	0.04	3.93	1.37	0.17	9.27	7.56	0.52***
Yes	66.1			4.18	1.63		5.01	9.87	
Age						*f*-value			*f*-value
Under 20 years	71.0	3.81	0.12	3.29	1.75	3.29*	6.86	8.03	0.76^ns^
20–29	67.1			3.96	1.50		7.71	9.20	
30–39	56.1			4.11	1.41		8.98	8.67	
40 or older	65.5			4.20	1.06		8.93	6.45	
Work status									
Full-time	56.4	4.05	0.12	3.99	1.21	1.40	10.11	6.98	5.23**
Part-time	68.0			4.00	1.57		8.54	6.64	
No work affiliation	69.8			4.27	1.60		5.21	10.69	
In education	65.4			3.73	1.56		7.58	8.09	
Living situation									
Living alone	64.7	6.58	0.15	3.65	1.61	4.25**	5.00	11.04	2.42*
Living with someone	73.3			3.47	1.67		7.98	7.57	
Living with children, no spouse	68.8			4.50	1.16		7.03	8.54	
Living with a spouse, no children	48.9			3.83	1.55		9.28	9.15	
Living with spouse and children	61.9			4.24	1.20		9.57	6.98	

* *p* < 0.05.

** *p* < 0.01.

*** *p* < 0.001.

### 4.3 Multiple linear regression analyses

The results of the twelve multiple regression analyses are displayed in Table 4. Taken together and controlling for all other variables, results showed that increased time spent playing FBR and being socially motivated to play predicted expanded networks for all participants (men, women, and those with or without a disability), with beta-values ranging from .29 to .46 (most substantial for socially motivated women).

**Table 4:** Multiple linear regression analyses. Separate analyses were conducted for each dependant variable by gender and disability status.

	Expanded network				Impact on life				Well-being			
	Male	Female	No disability	Disability	Male	Female	No disability	Disability	Male	Female	No disability	Disability
Total hours game time Fortnite (TGF)	.29**	.32**	.29***	.32*	.13	.05	.11	.35*	.02	−.01	−0.07	.15
Social motivation	.34***	.46***	.44***	.38*	.36***	.01	.21**	−.07	.00	.18*	0.08	.27
Achievement motivation	−.03	−.08	−.06	−.09	.03	−.04	.05	−.19	.10	−.06	0.02	.03
Novelty motivation	.02	−.13	.01	−.24	.02	.21*	.06	.14	−.02	.03	0.13	−.36*
Gaming with family and friends	−.14	−.10	−.15*	−.14	−.09	−.04	.00	−.16	−.06	.17	0.06	.05
Gender^a^	–	–	−.07	.16	–	–	−.08	−.14	–	–	−0.19*	.26
Age (40 or older ref.)												
Under 20 years	.08	.05	.21	−.31	−.11	.03	−.07	−.22	.00	−.31*	−0.13	.04
20 to 29	.12	.03	.08	−.12	.04	.05	.00	.24	.14	−.22	−0.01	−.41
30 to 39	−.02	−.08	.01	−.30	.01	.07	.04	.05	.15	−.14	0.08	−.15
Work status (fulltime ref.)												
Parttime	.14	−.11	−.04	.16	.07	.01	.02	.00	−.01	−.04	−0.02	−.02
no work affiliation	.06	−.11	.05	−.09	−.11	.16	.11	−.11	.00	−.30**	−0.06	−.38
in education	.19	−.04	.01	.22	.04	.11	.17	.05	.02	−.02	0.00	−.02
Living situation (Living alone ref.)												
Living with someone	.08	−.04	−.03	.09	−.10	−.13	−.14	.02	−.05	.26*	0.07	.27
Living with children. no spouse	.04	−.08	.04	−.25	−.03	.22	.11	.26	−.05	.15	0.04	.01
Living with spouse. no children	−.14	−.03	−.06	−.29	−.20	.15	−.06	.18	−.02	.29*	0.12	.27
Living with spouse and children	.14	−.14	.00	−.17	−.03	.15	.05	.41	−.07	.38**	0.08	.36
Total hours game time other games (TGG)	−.04	−.06	−.02	.02	.00	−.01	−0.04	−.02	−.14	−.10	−0.11	0.4
*N*	133	132	207	56	134	132	207	57	130	130	201	57
adj. R-sq	.35	.29	.33	.35	.16	.03	.07	.07	−.09	.18	.01	.04

^a^gender male = 0. Female = 1.

* *p* < 0.05.

** *p* < 0.01.

*** *p* < 0.001.

Time spent gaming FBR (having a disability, *β *= *.35*), being socially motivated to play (men *β *= *.36*, having no disability *β *= *.21*) and Novelty motivation (women, *β *= *.21*) predicted a positive impact on life. For women, social motivation predicted higher well-being (*β *= *.18*). Novelty motivation negatively predicted well-being for those having a disability (*β *= −*.36*). For men, the adjusted R-squared values are negative in the model predicting well-being, which may seem counterintuitive. However, adjusted R-squared can yield negative estimates for small R-squared values (54), indicating that the model has no predictive value. This phenomenon is observed only in the case of men, as the adjusted R-squared for the model explaining well-being is 0.18 for women.

## 5 Discussion

This study aimed to examine associations between online gaming, expansion of social network, and well-being. Specifically, we examined if time spent gaming, different types of motivation for playing FBR, and whom one game with are associated with expanded network, impact on life, and well-being. Moreover, we conducted gender and disability-specific analyses. We controlled for time spent gaming (other games than FBR), age, work status and living conditions. Overall, the results revealed different associations concerning social aspects of gaming in men, women, and those with and without a disability.

### 5.1 Expanded network by playing FBR

Recall that we asked whether participants had expanded their social network (formed new acquaintances and friends they met regularly offline or online) by playing FBR. In our sample, approximately 63 percent of the participants reported an expansion of their social network, and among participants with no work affiliation, the percentage was close to 70 percent. Being unemployed is associated with loneliness (55), and gaming helps expand one's network, which may indirectly reduce loneliness in this vulnerable group.

Furthermore, the main analyses showed that social motivation was the only gaming motivation associated with expanded social networks for both men and women (with and without a disability). Hence, individuals who were socially motivated to play FBR were the ones who also expanded their social networks. In a previous study, it has been argued that social motives might be of importance relating to social outcomes (56). If one enters a game with intentions to communicate, get to know others, and cooperate, one is perhaps more likely to develop friendships with co-players. Such reasoning is supported by findings showing that positive and pro-social conduct during gameplay improves friendship quality (57). Moreover, higher frequency and duration of whom one play with has shown to create stronger social bonds (58), which may explain why time spent playing FBR in our study was associated with an expanded social network. Those who spend more time playing might game with the same individuals over a period, thus developing deeper friendships. Being motivated by novelty and achievement had no association with participants’ networks in the current study. The findings presented herein support the notion that motivation for play matters when it comes to the social outcomes of gaming. Given the cross-sectional nature of this study, we cannot rule out the possibility that those who increased their network playing FBR were the ones who scored higher on social motivation.

### 5.2 FBR impact on life and well-being

Even though both men and women reported that playing FBR impacted their lives positively, as displayed in Table 2 (with mean scores above three for all), women evinced significantly higher scores on this variable than men. There were no significant differences between those with and without a disability. The main analyses revealed that for those with a disability, more time spent on FBR was associated with a perceived positive impact on life. There may be several ways to interpret this finding. First, a positive association between increased gaming time and the satisfaction of the three basic psychological needs has been previously found (27). It may be that individuals with a disability spend more time gaming to fulfil these needs, especially the need for relatedness, which is in line with the reasoning that individuals who have fewer opportunities to interact with others are perhaps the ones who particularly benefit from the social side of gaming (30). In our study, those with a disability spent more time gaming than those who did not have a disability (see Table 2), which may be because they have more time to game (many may be unemployed or not in school). However, if one struggles with mobility, social anxiety, depression, or other issues that somehow hinder one from participating in the “*offline* world”, gaming may represent an arena with easy access to other people. Such reasoning aligns with a study that showed that young men with a disability experienced that gaming provided engagement with peers through a “shared identity of a gamer” (25). Hence, while gaming, one's disability becomes invisible, and one participates on equal terms, which may partly explain the positive association between time spent gaming and the positive impact on life for this particular group of gamers.

Furthermore, social motivation was positively associated with the impact on life for men and those without a disability. Men generally have less extensive social networks and fewer friends than women (36). Being socially motivated to play FBR may compensate for these shortcomings and thus provide a subjective experience that FBR positively influences their lives. We do not have an explanation for why social motivation was associated with a positive impact on life in those participants without a disability. However, generally speaking, if one is socially motivated to play, one is perhaps more likely to fulfil the need to feel connected with others (24), and satisfy the need for relatedness (26), which, in turn, may influence how one evaluates how the game has impacted one's life. In addition, novelty motivation was associated with a positive impact on life for women only. Being motivated by novelty in FBR includes getting new skins, avatars, and discovering new places on the map, thus experiencing new things in the game. Why this motivation impacts women's lives positively is hard to say. One explanation may be that women do more household chores than men (59): Thus, being able to enter another world, discover new places, get special abilities, and have great costumes can provide an outlet from everyday routines and thus positively impact life. The same argument may explain why social motivation was associated with increased well-being for women. Gaming may be an arena where women can connect with others in a busy everyday life as it can be done from home. Thus, socially motivated women may fulfill their need to belong through gaming (24), which may even result in higher levels of well-being. Finally, being motivated by novelty was associated with lower well-being in those with a disability. Some studies show that gamers create avatars with better “assets” than players have in real life (60, 61). According to the self-discrepancy theory (62), the greater the gap between the actual and ideal selves, the more distress the individual will suffer. Additionally, as shown in a previous report (38) using the same sample as the current study, individuals receiving disability pensions spent the most money on FBR, including skins, emotes and accessories, compared to those with work affiliations. Thus, by acquiring better assets in FBR, disabled who are novelty-motivated may spend a disproportionate amount of money, leading to a significant disparity between the actual self and the ideal self, represented by the avatar in FBR, and potentially leading to economic concerns that could explain lower well-being in this particular group.

## 6 Limitations

In the present study, we focused on one game, Fortnite Battle Royale, and accordingly, the findings are only partly generalizable to other games and other gaming populations. However, scholars have called for more game-specific studies; “if one wants to know what the effects of video games are, the devil is in the details” (63, p. 763). Furthermore, another limitation is that we applied a convenience sample where participants were recruited through various digital channels, and our participants may not be representative of FBR players in general. Additionally, given the cross-sectional design, we cannot ignore the possibility that the direction of effects could be reversed in some of our findings. For example, it may be that women who score higher on well-being are the ones who are most socially motivated to game. Moreover, our study is based on self-reports, which sometimes can prove unreliable. For instance, players often lose track of time while gaming (64) and, thus, can under or over-report time spent gaming. Applying additional measures, such as objective game play measures could have enhanced the reliability for the time spent gaming variable. Additionally, participants may have answered in a socially desirable manner (65). Another limitation concerns two of the outcome measures applied in the study, “expanded network” and “impact on life”. These are single items developed by the authors; thus, there is some uncertainty regarding the validity and reliability of these measures.

## 7 Conclusion

The current study addressed the social aspects of gaming in a sample of adult gamers of FBR. Findings showed that time spent gaming and social motivation to play FBR were associated with expanded social network for all participants. Also worth noting was the finding showing that more time spent gaming was associated with a perceived positive impact on life for those with a disability. These findings are promising, and further investigations in this area are needed. It would be worthwhile to investigate the same issues on other groups at risk of marginalization longitudinally to examine whether gaming may enhance their social participation and general well-being.

## Data Availability

The raw data supporting the conclusions of this article will be made available by the authors, without undue reservation.
